# Docking and virtual screening of novel inhibitors for mono-ADP-ribosylating toxins

**DOI:** 10.1186/1758-2946-3-S1-P38

**Published:** 2011-04-19

**Authors:** M Scharfe, B Maurer, K Aktories, M Jung, W Sippl

**Affiliations:** 1 Institut für Pharmazie, Martin-Luther-University of Halle, Germany; 2 Institut für Pharmazeutische Wissenschaften, Albert-Ludwigs-University of Freiburg, Germany

## 

**Figure 1:**
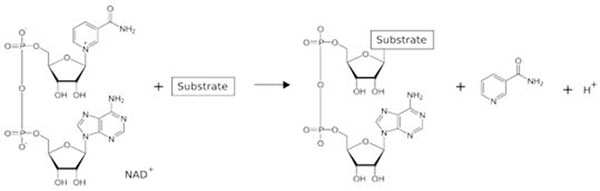


ADP-ribosyltransferases (ADP-RTs) are a family of enzymes secreted by pathogenic bacteria. They catalyse the hydrolysis of NAD+ and the transfer of the ADP-ribosyl group onto specific target proteins [1,2]. Figure 1

Although ADP-RTs are important drug targets, only few inhibitors are known so far. The high selectivity of these inhibitors suggest different mechanism of binding to the ADP-RTs active sites. To explain the structural differences, we started a systematic program towards the development of new ADP-RT inhibitors. This is based on multiple virtual screening experiments (e.g. docking, pharmacophore searching and binding free-energy calculations) and the development of an in vitro assay for ADP-RTs. Active compounds identified in the first screening round are the basis for further in silico studies and optimisation steps. The aim of the current work is the discovery of novel drug-leads and the formulation of structure-function relationships which can explain the selectivity of ligand binding to the NAD+ pocket. This is interesting from a drug discovery perspective, as many enzymes utilizing NAD+ are valid or potential drug targets.
